# Is Aneuploidy a Consistent Marker for Malignant Transformation Risk in Oral Lichen Planus?

**DOI:** 10.1007/s12105-025-01779-x

**Published:** 2025-05-08

**Authors:** Aya Pessing-Shabi, Ayelet Zlotogorski-Hurvitz, Noam Yarom, Ilana Kaplan, Luba Trakhtenbrot, Abraham Hirshberg

**Affiliations:** 1 Department of Oral Pathology, Oral Medicine and Maxillofacial Imaging, School of Dental Medicine, Faculty of Health and Medical Sciences, Tel Aviv University, Tel Aviv, Israel; 2 Oral Medicine Unit, Sheba Medical Center, Tel-Hashomer, Israel; 3 Cancer Research Center, Sheba Medical Center, Tel-Hashomer, Israel; 4 Oral Medicine Unit, Tel Aviv-Sourasky Medical Center, Tel Aviv, Israel; 5 Department of Oral and Maxillofacial Surgery, Beilinson Hospital, Rabin Medical Center, Petah-Tikva, Israel; 6 Department of Oral and Maxillofacial Surgery, Kaplan Medical Center, Rehovot, Israel; 7 School of Dental Medicine, Faculty of Health and Medical Sciences, Tel Aviv University, Tel Aviv, Israel

**Keywords:** Aneuploidy, Aneuploid Cells (ACs), Oral Lichen Planus (OLP), Oral Potentially Malignant Disorder (OPMD), Oral Squamous Cell Carcinoma (OSCC)

## Abstract

**Background:**

Numeric chromosomal imbalance, known as aneuploidy, is linked to both malignant and potentially malignant epithelial lesions. Aneuploidy has also been investigated in oral potentially malignant disorders (OPMDs) due to its high incidence in head and neck cancers, particularly in oral squamous cell carcinoma (OSCC). The study aimed to evaluate the potential of aneuploidy, a marker of chromosomal imbalance, as a prognostic tool for assessing malignant transformation risk in oral lichen planus (OLP) patients.

**Methods:**

Fluorescent in situ hybridization (FISH) analysis targeting centromeric probes for chromosomes 2 and 8 was conducted on samples from 245 patients, with follow-up in 135 cases.

**Results:**

Aneuploid cells (ACs) were detected in 73 patients (29.8%); 24 (32.9%) exhibited non-diploid cells in a normal looking mucosa. Only 2 (0.8%) patients developed OSCC during the follow-up. Among the 135 followed, 11 (8.1%) were positive for Acs in both samples, 15 (11.1%) were were negative initially but positive later. In contrast, 3 patients (2.2%) were initially positive but later negative.

**Conclusion:**

These results indicate a low malignant transformation rate (< 1%), despite a high rate of aneuploidy. These also demonstrate variability in aneuploidy results over time. The dynamic nature of aneuploidy observed suggests that it may not be a reliable predictive tool for malignant transformation in OLP.

## Introduction

Oral lichen planus (OLP) is a relatively common inflammatory mucocutaneous disease, characterized by a chronic pattern of relapses and remissions [1, 2]. It is a T-cell-mediated immune condition of unknown etiology with a role of immune dysregulation in the pathogenesis, involving both antigen-specific and non-specific mechanisms with multiple factors influencing immune function such as genetic, environmental or psychological [3, 4].

The prevalence varies between 0.1 and 3% of general population, depending on geographic region, with an overall pooled estimated prevalence of 0.9% [5, 6].

OLP is most frequently a disease of the middle-aged and elderly with a peak incidence in the 30–60 years range with a female predominance of 2:1. Although rarely, younger adults and children may be affected [7, 8].

The oral sites most frequently involved are buccal mucosa, tongue and gingiva. OLP lesions are usually multifocal, symmetrical and bilateral. The lesions are classified into six clinical variants: reticular, papular, plaque-like, atrophic, erosive and bullous, with reticular, erosive and atrophic being the most common manifestation. Oral lesions may be chronic in nature, remitting and relapsing with varying degrees of morbidity. Although they are often asymptomatic, atrophic, erosive and/or ulcerative lesions might be painful [9, 10].

The World Health Organization (WHO) [11] classified OLP as a potentially malignant condition, later updated to oral potentially malignant disorder (OPMD) [12]. Patients diagnosed with OPMDs may have an increased lifetime risk of developing oral malignancy. The majority of OPMDs may not transform into carcinoma, but rather provide an area of altered epithelium in which cancer is more likely to develop. These individuals could carry an endogenous risk factor, rather than being exposed to an environmental factor such as smoking tobacco and alcohol abuse [12, 13]. The actual risk of transformation in OLP varies widely, ranging from 0.04 to 1.74% annually. The erosive and/or atrophic types and tongue involvement carry a significantly higher risk of malignant transformation [14, 15].

Numeric chromosomal imbalance, referred to as aneuploidy, has been associated with malignant and potentially malignant epithelial lesions. The presence of aneuploidy in tumors at an early stage and the implementation of DNA content analysis as a useful marker for determination of prognosis in these lesions has been thoroughly investigated [16, 17]. This bio-marker tool has been also investigated in OPMDs since the incidence of aneuploidy is high in head and neck cancer, and particularly in oral squamous cell carcinoma (OSCC). Recently, several studies have used this bio-marker as a prognostic method for malignant transformation of OPMDs but only a few cytogenetic studies have been conducted in OLP [17, 18]. In our previous studies we evaluated the presence of chromosomal numerical aberrations in cells collected by brush sampling from persons with OLP by combined morphological and fluorescence in situ hybridization (FISH) analysis [18–20]. We have found aneuploid cells in about a quarter of the participants. Aneuploid cells were also detected in the normal-looking mucosa. In a follow-up study, 3 out of 57 patients have developed oral cancer within an average follow-up period of 32 months. Over 10% aneuploid cells (ACs) were found in the brush sample taken from the affected oral sites in these patients [19, 20].

In the present study we aimed to determine whether cell ploidy sampled by oral brush from OLP patients can be a useful prognostic tool to determine whether a patient should be under close follow-up, at least twice a year, or whether he can be considered low risk and submitted to less frequent routine checkups.

## Materials Methods

### Study Population

The study group included 268 patients with OLP, collected from three medical centers: The Sheba Medical Center, Rabin Medical Center and Tel Aviv Sourasky Medical Center. All patients were referred to the Oral Medicine Clinics, for the diagnosis and management of OLP. In all cases, the clinical diagnosis of OLP was confirmed by histopathologic examination demonstrating hyperkeratosis, degeneration of the basal layer, and subepithelial lymphocytic band-like infiltrate based on the modified WHO diagnostic criteria [11]. The exclusion criteria comprised individuals under 18 years old and those with systemic conditions mimicking OLP such as Graft versus Host Disease (GVHD), Lupus Erythematosus, etc. Data including age, gender, medical history, habits (alcohol or tobacco consumption), sites of OLP involvement and clinical type of OLP (reticular, atrophic, and erosive) was collected. Information about the use of corticosteroids treatment (topical or systemic) was also retrieved. Thirty-three healthy patients with normal-looking oral mucosa served as the control group.

Consent was obtained from all participants in accordance with a protocol approved by the Institutional Review Board for Clinical Studies and by the Ministry of Health for the use of genetic material. The total follow-up period was 5 years. Data of malignancy was obtained from the Gertner Institute of Epidemiology and Health Policy Research (Sheba Medical Center, Tel-Aviv University).

### Samples Collection

Oral samples were collected with a disposable 5 segments heads brush with high density of well-defined soft, flexible plastic hairs (Orcellex^®^ Brush, Rovers Medical Devices, The Netherlands). The brush was small and rigid enough to allow easy and non-painful collection of cells from all layers of the oral epithelium. The sample was placed in a methanol-based, buffered preservative solution (ThinPrep^®^ CytoLyt^®^ Solution, Marlborough, MA, USA).

Two samples were taken from each participant: 1- from an area affected by OLP; 2- from a normal-looking mucosa at a different oral site, preferably, whenever possible, at the contralateral site. Only samples that had more than 50 cells collected for analysis were included in the study. Samples were collected again 1–2 years later. In the control group, consisting of 33 participants, a single sample was taken from the buccal mucosa of each individual.

### Samples Preparation and FISH Analysis

The samples were prepared by standard procedures that included cytospin, fixation, co-denaturation and hybridization [21]. Dual-color FISH with centromeric probes for chromosome #2 labeled with Spectrum Green and chromosome #8 labeled with Spectrum Orange (Vysis, Downer’s Grove, IL, USA) was performed. Cells with fluorescent signals were scanned manually and captured as target nuclei. In normal diploid nuclei we expect to see hybridization pattern with 2 red and 2 green signals. A sample was determined to be aneuploid if one of the probes showed more than 2 signals and when the number of aneuploid cells was 0.5% and higher of the total number of analyzed cells.

The percentage of cells with more than 2 signals of chromosomes #2 and #8 from the entire cell population was calculated for each case.

The selection of chromosomes #2 and #8 was based on the results of our previous studies, in which we used these chromosomes successfully to detect aneuploid cells in OLP, oral leukoplakia, and OSCC [18–20]. Additionally, broader literature supports their role in chromosomal instability in premalignant and malignant oral mucosal lesions. Both chromosomes harbor genes involved in tumorigenesis across various malignancies, with MYC and MYCN amplification playing a critical role in oncogenesis [20, 22–24].

### Statistical Analysis

The statistical analysis was conducted on matched data at the patient level. For each patient, two initial measurements were obtained: one from the affected side (test) and one from the contralateral side (control). The main outcome was the number of aneuploid cells among total investigated cells. Correspondingly, we applied negative binomial regression with bootstrap estimation of variance for panel data, considering patient as a panel. The analysis was performed on data from the initial probe measurements and included follow-up probes taken after one year. For patients missing follow-up probes after one year, the observations were imputed as zero tested cells with zero aneuploid cells. Changes within one year were also assessed for patients with measurements taken at both time points. Cut-point of 0.5% and above was considered positive for 1st and the 2nd tests.

All statistical tests were two sided, with significance defined at p-values less than 0.05. The analysis was performed using STATA 16 SE software.

## Results

The final study group included 245 patients with more than 50 cells in each sample.

The mean age was 63.5 (range 23–93 years) with 1:3 male-to-female ratio. Clinical and demographic results are presented in Table 3 (appendix).

Aneuploid cells over 0.5% of the examined cells were detected in 73 cases (29.8%) (Table 1), with an average of 131 cells examined in each sample, and a mean result of 5.7% aneuploid cells. This was referred to as the positive group. Patients with less than 0.5% aneuploid cells were considered negative.

**Table 1:** The positive group (*N* = 73)

mean AC%	range AC%	No. of cells examined (average, range)	3r,2 g	3 g,2r	3 g,3r	4 g,2r	3r,3 g	3 g,2r
5.7%	0.5–80%	131 (50–300)	24 (32.9%)	14 (19.2%)	10 (13.7%)	10 (13.7%)	11 (15.1%)	10 (13.7%)

AC- aneuploid cells

g-green. r-red

*4r,4 g−2; 4r,3 g−3; 3r,4r−1; 3 g,4 g−3

**Signal 4 included 4 and above. There were 8 samples with more than 4

There was no statistical difference in gender and age between the positive and negative groups.

There was little information of alcohol and tobacco consumption with no differences between the two groups. The distribution of the clinical form of OLP was similar in both groups, with the reticular form being the most common, close to 60% in both groups.

No correlation was found between gender, smoking habits, clinical presentation, medical history and the proportion of aneuploid cells detected in the positive group.

The control group included 33 subjects. The mean age was 52.1 yrs, with a 1:2 male-to-female ratio. Three people with positive results (9%)- 2 with 0.5% aneuploid cells and one with 5%. The frequency of aneuploidy was significantly lower in the control group (*p* = 0.012). No significant statistical differences were found in all other parameters according to Fisher’s exact test.

Of the 73 positive cases, 24 (32.9%) exhibited non-diploid cells in a normal looking mucosa and 17 (23.3%) were positive in both normal and affected mucosa.

### Follow Up

Among the 268 participants, only 135 underwent testing twice, with an average interval of 15 months between tests, ranging from 10 to 33 months. The proportion of males was 28.15%, with no significant difference between those tested once and those tested twice.

Nine (3.7%) patients developed non-oral cancer during follow up of which 2 were positive for aneuploidy in the first sample. Only 2 (1.48%) patients developed OSCC (Table 2), one case in the tongue and the other in the gingiva. The first patient was diagnosed with OSCC 41 months after the first sample was taken. Two samples were taken from this patient, 14 months apart, both positive with 0.5% aneuploid cells in the first sample, and 1% in the second sample. In the second sample, aneuploid cells were found in the normal appearing mucosa and negative in the affected side. The OLP clinical presentation was of atrophic type. The second patient was diagnosed with OSCC in the gingiva 22 months after the first sample. Second sample was taken after 13 months, no aneuploid cells were tested in both samples. The OLP clinical presentation was of erosive type.

**Table 2:** Detailed information regarding the two patients with oral malignant transformation

Case	1		2	
Age (y), Sex	82, M		73, F	
Tobacco	No		No	
Location	Tongue		Gingiva	
Type	SCC		SCC	
Time to transformation (months)	41		22	

Clinical type	ALP	Normal looking	ELP	Normal
First test (AC%)	0.50%	0	0	0
No of cells	200	100	50	50
Signal	4 g,2r			
Second test (AC%)	0%	1%	0	0
No of cells	100	100	70	200
Signal		4 g,2r	-	

*AC- anaeploidy cells, SCC- squamous cell carcinoma, ALP- atrophic lichen planus, ELP- erosive lichen planus, g-green, r-red

## Discussion

Oral cancer ranks as the 16th most prevalent cancer globally [25], comprising 377,713 new cases and 177,757 deaths in 2020 [26], with a 5-year survival rate ranging from 50 to 66% [27]. In 2020 the WHO classified Oral Lichen Planus (OLP) as an Oral Potentially Malignant Disorder (OPMD) [28].Recent meta-analysis has a substantiated low malignancy rate, with the transformation rate in OLP ranging from 0.44 to 2.28% [29]. However, it is advisable to exercise caution and implement long-term follow-up for patients with OLP, particularly focusing on those presenting with atrophic/erosive/ulcerative manifestations of the disease, lesions on the tongue, and individuals with additional risk factors, such as tobacco and alcohol use [15]. When evaluating the malignant risk associated with OLP, it is essential to distinguish between OLP and Oral Lichenoid Lesions (OLLs), which are considered clinical and histological counterparts of classical OLP [11]. Unlike the idiopathic nature of OLP, OLLs are often associated with identifiable triggering factors [30]. Furthermore, differentiating between OLP and OLL is crucial, as OLLs carry a slightly elevated risk (1.88%) of progressing to oral cancer [15]. Thus, given the wide spectrum of clinical presentation, and the risk for transformation, there is a need for the development of a practical prognostic tool to identify patients at risk.

Aneuploidy is a deviation from the normal DNA or chromosomal complement in a cell, caused by chromosomal instability, which can manifest as duplication or deletion of chromosomes or parts of chromosomes, and it is a fundamental process in dysplasia and carcinogenesis [31].

In head and neck squamous cell carcinoma (HNSCC) and specifically in oral squamous cell carcinoma (OSCC), a markedly elevated incidence of aneuploidy has been observed [32, 33]. These highlights the potential of this molecular biomarker as a tool to evaluate the risk of malignant transformation. The need to identify the high-risk patient group and subject them to frequent follow-up to provide early diagnosis of oral cancer, led to an ongoing search for molecular and genetic methods that could detect this population.

The present study results, conducted in collaboration with three major medical centers with a wide range of OLP patients, revealed a transformation rate of 0.8%, a result which is in consistent with recent reports from other studies [29, 34]. The routine follow-up protocols for OLP patients executed by specialists in oral medicine in all three medical centers, provides strong validation for the demonstrated transformation rate.

In the present study, 135 patients were tested at 2 time-points, of which 11 (8.1%) were positive in both samples, 15 (11.1%) were negative in the first sample but positive in the second. In contrast, 3 patients (2.2%) were positive in the first sample but negative in the repeated test. These findings indicate that there is instability in results over time. It is important to emphasize that the total number of cases with confirmed malignant transformation (0.8%) was significantly lower than those with aneuploid result in either a single or in both the samples tested. The dynamic nature of aneuploidy in the present study poses significant implications for the practical application of aneuploidy assessment in the clinical management of OLP. The non-consistency observed in aneuploidy states over time suggests that a single-point assessment may not accurately reflect the long-term risk profile of an individual. Such variability challenges the fundamental assumption that aneuploidy is a stable and irreversible precursor to carcinogenesis.

The unique methods in the present study which tested each patient at two different time points, enabled us to conclude that aneuploidy is not necessarily a constant and stable condition whereas previous studies performed at only a single time point demonstrated the relative frequency of aneuploidy without providing information on its stability follow up.

Several factors contribute to the challenges associated with using aneuploidy as a predictive tool. Aneuploidy can arise not only from exposure to carcinogens but also from natural aging processes in all tissues [35]. Additionally, the effectiveness of FISH as a diagnostic tool is limited by the challenge of establishing definitive thresholds for identifying aneuploidy, as well as the presence of mixed populations of both normal and potentially multiple clones of dysplastic cells, which can exhibit varying copy numbers. It can be particularly challenging to differentiate between low copy number gain in abnormal cells and normal diploidy in neighboring cells. Furthermore, although methods for detecting DNA aneuploidy claim to detect minimal changes in nuclear DNA, their sensitivity for clinical samples remains undefined. OPMDs exhibit clonality and comprise mixed populations of aneuploid and others apparently diploid, at a microscopic level. While aneuploid clones may dominate in certain lesions, others may contain limited numbers of aneuploid cells, making their detection challenging [36]. Generally, diploid DNA results demonstrate a very high negative predictive value. However, predictive values vary across studies, likely due to methodological differences and the diversity of populations studied [37]. In high-risk populations, the negative predictive value diminishes due to a higher proportion of lesions with transformation potential, such as those adjacent to carcinoma or in untransformed OPMD used as a control population [38, 39]. Previous studies have suggested that DNA aneuploidy may possess predictive value in OLP, but results of the present study do not support this [40, 41]. Challenges in definitively diagnosing OLP from other OPMDs may contribute to excessive reported rates of aneuploidy in some studies, which appear inconsistent with OLP’s extremely low transformation rate [29, 42].

In conclusion, for the population studied, transformation rate in OLP was found to be less than 1%, over long-term follow-up periods. Despite the detection of chromosomal instability in a significant proportion of these patients, it did not prove to be predictive enough to serve as a practical tool for risk assessment. However, due the relative rarity of the condition in the populations, the present study is still one of the largest studies published in this field.

Some of the limitations in our study include relatively uniform population and number of cases included. Further studies with larger number of subjects are required to better understand the correlation of malignant transformation in OLP patients and the spectrum of chromosomal instability, and sensitivity of aneuploidy as a predictive marker.

### Appendix

**Table 3:** Demographic and clinical data

	Positive Group, *N* = 73	Negative Group, *N* = 172	Total, *N* = 245	Control *N* = 33	Statistical Significance
Mean age (yrs, range)	61.93 (28–93)	64.11 (23–90)	63.46 (23–93)	52.12 (33–81)	NS
male (%)	22 (30.14%)	44 (25.58%)	66 (26.94%)	13 (39.39%)	NS
female (%)	51 (69.86%)	128 (74.42%)	179 (73.06%)	20 (60.61%)	NS
smoking (%)	17 (23.29%)	25 (14.53%)	42 (17.14%)	1 (3%)	NS
Alcohol use (%)	2 (2.74%)	4 (2.74%)	6 (2.45%)	2 (6.06%)	NS
Reticular lichen planus (%)	43 (58.91%)	99 (57.55%)	142 (57.96%)	NR	NS
Erosive lichen planus (%)	5 (6.84%)	15 (8.72%)	20 (8.16%)	NR	NS
Atrophic lichen planus (%)	25 (34.25%)	58 (33.73%)	83 (33.88%)	NR	NS
Systemic disease	24 (32.88%)	59 (34.30%)	83 (33.88%)	NR	NS
Metabolic disease	12 (16.44%)	32 (18.60%)	44 (17.96%)	NR	NS
Autoimmune disease	2 (2.74%)	7 (4.01%)	9 (3.67%)	NR	NS
Malignanacy	13 (17.80%)	27 (15.69%)	40 (16.33%)	NR	NS
Oral cancer before	1 (1.37%)	6 (3.49%)	7 (2.86%)	NR	NS
Steroids use	9 (12.33%)	22 (12.79%)	31 (12.65%)	NR	NS

NR-non relevant

NS- not significant

## Data Availability

No datasets were generated or analysed during the current study.

## Code Availability

No custom code was used for this work.
